# Current perspective of new anti-Wolbachial and direct-acting macrofilaricidal drugs as treatment strategies for human filariasis

**DOI:** 10.3205/id000079

**Published:** 2022-03-30

**Authors:** Alexandra Ehrens, Achim Hoerauf, Marc P. Hübner

**Affiliations:** 1 Institute for Medical Microbiology, Immunology and Parasitology, University Hospital Bonn, Germany; 2 German Center for Infection Research (DZIF), partner site Bonn-Cologne, Bonn, Germany

**Keywords:** filariae, onchocerciasis, Wolbachia, doxycycline, ABBV-4083, emodepside, oxfendazole, rifampicine, Litomosoides sigmodontis, Onchocerca volvulus

## Abstract

Filarial diseases like lymphatic filariasis and onchocerciasis belong to the Neglected Tropical Diseases and remain a public health problem in endemic countries. Lymphatic filariasis and onchocerciasis can lead to stigmatizing pathologies and present a socio-economic burden for affected people and their endemic countries. Current treatment recommendations by the WHO include mass drug administration with ivermectin for the treatment of onchocerciasis and a combination of ivermectin, albendazole and diethylcarbamazine (DEC) for the treatment of lymphatic filariasis in areas that are not co-endemic for onchocerciasis or loiasis. Limitations of these treatment strategies are due to potential severe adverse events in onchocerciasis and loiasis patients following DEC or ivermectin treatment, respectively, the lack of a macrofilaricidal efficacy of those drugs and the risk of drug resistance development. Thus, to achieve the elimination of transmission of onchocerciasis and the elimination of lymphatic filariasis as a public health problem by 2030, the WHO defined in its roadmap that new alternative treatment strategies with macrofilaricidal compounds are required. Within a collaboration of the non-profit organizations Drugs for Neglected Diseases initiative (DND*i*), the Bill & Melinda Gates Foundation, and partners from academia and industry, several new promising macrofilaricidal drug candidates were identified, which will be discussed in this review.

## Filariasis

Filarial nematodes can cause debilitating diseases like lymphatic filariasis (LF) and onchocerciasis. LF is caused by *Wuchereria bancrofti*, *Brugia malayi* or *Brugia timori*, which can lead to lymphedema in the extremities (elephantiasis) and/or hydrocele in the scrotum of men [1], [2]. The nocturnal mosquito vector (*Mansonia, Anopheles, Culex, Aedes*) transmits infective L3 larvae to humans, which migrate to the lymphatics and mold into adult worms. The progeny, so-called microfilariae (MF), are released into the peripheral blood, where they can be taken up again by the mosquito. LF is mainly found in sub-Saharan Africa and South-East Asia and it is estimated that in 2017, 65 million people were infected with LF, while 19.42 million people suffered from hydrocele, and 16.68 million people from lymphedema [3], [4].

The causative agent for onchocerciasis is *Onchocerca volvulus*, and the disease can lead to vision impairment and blindness as well as severe dermatitis. Thus, onchocerciasis is often referred to as river blindness; and the disease is mainly found in sub-Saharan Africa; and remaining foci in Yemen, Venezuela and Brazil exist [2], [5], [6]. The infective L3 larvae are transmitted by black flies (*Simulium*), and the adult worms reside in subcutaneous nodules, while the MF migrate through the skin and occasionally the cornea [2]. In 2017, onchocerciasis affected about 21 million people with 14.6 million people presenting skin diseases and 1.15 million people suffering from vision impairment [3], [4], [6].

The pathology in those diseases is induced through different mechanisms. While lymphedema initially occurs as an immunological consequence to the adult worms, which leads to the dilatation of the lymphatics and a leakage of the endothelial cell-cell junctions, skin manifestations and vision impairment in onchocerciasis are a result of an inflammatory immune response to the dying MF [7], [8]. Most human-pathogenic filarial nematodes live in an endosymbiotic state with *Wolbachia* bacteria, which provide important factors for worm development, growth and reproduction [9]. Release of these *Wolbachia* from dying MF provokes inflammation in the skin leading to rashes, itching, lesions in the skin or the cornea of the eye, and thus over time to vision impairment in onchocerciasis patients [10], [11], [12].

## Current treatment strategies and their limitations

Standard drugs used for mass drug administration (MDA) include ivermectin (IVM), albendazole (ALB), and diethylcarbamazine citrate (DEC) [1], [7]. IVM is a macrocyclic lactone with a microfilaricidal effect that temporarily inhibits the embryogenesis of adult female worms, which interrupts microfilaremia for up to 6–12 months and thus limits the transmission of the diseases [13]. Similarly, the piperazin derivate DEC also targets the MF, leading to a temporary inhibition of the embryogenesis of female worms. The carbamate benzimidazole ALB is another drug used in combination for MDA. Its mode of action is the inhibition of the polymerization of β-tubulin and microtubule formation in helminths. Thus, this broad-spectrum anti-helminthic drug is not specifically used to treat filariasis but is active against other nematodes and cestodes [14], [15]. On its own, ALB has only little effect on MF and adult filariae [16].

For the control of onchocerciasis, the WHO recommends MDA of IVM on an annual or bi-annual basis. Treatment of LF is done with a combination of IVM and ALB in sub-Saharan Africa in areas that are co-endemic for onchocerciasis and with a triple therapy of DEC, IVM, plus ALB in regions non-endemic for onchocerciasis and loiasis [17], [18], [19]. MDA programs that have existed for up to 40 years now, have reduced the prevalence of LF. Seventeen countries have achieved elimination, and five additional countries have stopped MDAs and are now under surveillance of having eliminated LF, but 50 countries are still endemic for LF and require further MDA treatments [20]. Large-scale treatments in Latin America already successfully led to elimination of onchocerciasis in Colombia and Ecuador in 2013 and 2014, respectively. Furthermore, onchocerciasis transmission was stopped in Mexico and Guatemala in 2011. Thus, 11 out of 13 foci in the Americas have stopped MDA, while Brazil and Venezuela still require MDA treatment in few foci [21].

However, there are certain limitations for the current treatment strategies against onchocerciasis. The triple therapy (IVM, ALB, and DEC) used to treat LF may cause serious adverse events in patients of loiasis, another filarial disease caused by *Loa loa*, and onchocerciasis patients [5], [22], [23], [24], [25], [26], impairing the implementation of the triple therapy in areas co-endemic of those diseases. Furthermore, current treatment strategies are restricted to temporarily inhibiting the embryogenesis of adult female worms and having a microfilaricidal effect. Thus, the chemotherapeutics used lack a pronounced macrofilaricidal, i.e. adult worm killing, efficacy. Therefore, repeated annual or bi-annual drug administrations are required for the reproductive life-span of the adult worms, which is up to five years for LF and up to 15 years for onchocerciasis [1]. Moreover, modeling studies suggest that the triple therapy is in particular valid in clearing filariasis in areas with high prevalence, while it loses its cost efficiency in areas with low prevalence [17]. The WHO roadmap on Neglected Tropical Diseases 2021–2030 defined as a main target the elimination of LF as a public health problem and to stop the transmission of onchocerciasis by 2030 via mass drug administrations (MDA) and vector control [27], [28], [29], [30].

Recently, the FDA approved moxidectin as a treatment for onchocerciasis. Moxidectin belongs to the same milbemycin class of macrocyclic lactone as IVM. Similar to IVM, moxidectin is a potent microfilaricide and inhibits the filarial embryogenesis. However, inhibitory effects of moxidectin are superior to IVM, providing a complete clearance of MF, which is maintained for a longer period of time compared to IVM [31], [32].

Thus, a clinical study has shown that a single moxidectin treatment reduced the skin MF to 0.6 per mg skin after 1 year, in comparison to a single IVM treatment which resulted in 4.5 MF/mg of skin. Moreover, the proportion of patients without detectable skin MF was higher (38%) in the group treated with moxidectin than in the group receiving IVM (2%). Importantly, 98% of patients treated with moxidectin had no detectable MF within the eyes at 18 months post treatment. IVM treatment resulted in no detectable eye MF in 90% of the patients after 18 months [32]. This reduced MF load in moxidectin-treated patients is suggested to be the result of an enhanced embryostatic effect [33]. Moxidectin’s safety profile was comparable to IVM, and rapid MF death can result in adverse events after moxidectin as well as after IVM treatment [31], [32]. Due to moxidectin’s superior activity against MF in comparison to IVM, it may accelerate elimination of onchocerciasis, and modeling studies suggest that annual moxidectin administration could replace bi-annual IVM MDA in areas with persistent transmission [33]. However, IVM entails the risk for severe adverse events in patients with heavy *L. loa* microfilarial loads and thus, implementation is limited in *L. loa* co-endemic areas. Even though no clinical trials to evaluate the safety of moxidectin treatment in loiasis co-endemic areas have been conducted yet, it is expected that, similar to IVM, moxidectin treatment could have the same limitation. Thus, moxidectin could replace IVM treatment and reduce transmission as well as infection rates. However, it remains to be determined whether a microfilaricidal drug that lacks a strong macrofilaricidal efficacy is sufficient to achieve the goals of the WHO roadmap to stop the transmission of onchocerciasis by 2030. IVM MDA has been applied for almost 40 years and led to significant achievements in reducing onchocerciasis transmission. This success of IVM is closely linked to the commitment of Merck (MSD) to provide IVM free of charge, which is not the case for moxidectin, and will therefore challenge its broader use and success in endemic regions.

Thus, the identification of an effective macrofilaricide, which can be administered over a short period of time, could support the elimination of filariasis by mopping up residual foci and for case management [34], [35].

## Endosymbiotic Wolbachia bacteria as targets for anti-filarial therapy

Screening drugs against the *Wolbachia* endosymbionts of filariae is a validated approach to identify new macrofilaricidal compounds [36]. Most human-pathogenic filariae, such as *W. bancrofti* [37], *B. malayi* [38], *B. timori* [39], *O. volvulus* [9], *Mansonella perstans* [40], [41], and *Mansonella ozzardi* [42] contain endosymbiotic *Wolbachia*, while *L. loa* lacks *Wolbachia* bacteria [43].

*Wolbachia* bacteria have also been found in filarial nematodes of animals, including filariae infecting cattle (*Onchocerca ochengi*, *Onchocerca gutturosa* and *Onchocerca lienalis*) [44], [45], [46], cats *(Brugia* *pahangi*) [47], [48], dogs (*Dirofilaria immitis* [47], [49], *Dirofilaria repens* [50]), and rodents (*Litomosoides sigmodontis*) [51]. Furthermore, in some animal filariae *Wolbachia* are absent, like in the rodent filaria *Acanthocheilonema viteae* [51]. *Wolbachia* are rickettsia-like endobacteria and are transmitted transovarially to the next generation. Thus, they are found in oocytes, all larval stages, the lateral chord and hypodermis of adult worms, but never in the male genitals [52].

The endosymbiotic relationship between *Wolbachia* and filariae is created through the exchange of certain proteins, which are essential for growth, survival and reproduction of the filariae, and allow the treatment of *Wolbachia*-containing filariae with antibiotics [53].

For *B. malayi* and their endosymbionts (*w*BM) it was shown that *w*BM had a highly reduced genome. *w*BM are able to synthesize purines and pyrimidines *de novo*, but lack genes for the synthesis of other amino acids except for *meso*-diaminopimelate, an amino acid required to produce peptidoglycan [38], [54], [55]. Moreover, the *w*Bm genome encodes for the synthesis of riboflavin, flavin adenine dinucleotide, and heme [38]. However, these genes are only partly or completely absent in the nematode genome and thus, *Wolbachia* may provide the nematode with heme [56], riboflavin, flavin adenine dinucleotides, as well as nucleotides [57], and may also be a source of ATP during energy-intensive larval-stage development with high cell division such as development and embryogenesis [58].

Due to the close relationship and the dependency of nematode and endosymbiont, targeting *Wolbachia* by antibiotics results in the inhibition of worm development and fertility.

### Doxycycline as first anti-Wolbachial drug

Initial murine studies using the filarial nematode *L. sigmodontis* showed that tetracycline depletes *Wolbachia* bacteria from the filariae, which leads to female worm sterility, inhibited embryogenesis, and over time clearance of microfilaremia [51], [59]. Moreover, using *O. ochengi*, *B. pahangi* and *D. immitis* models, a macrofilaricidal effect for tetracycline was confirmed [45], [47]. Doxycycline, a second-generation tetracycline antibiotic, was subsequently used in first clinical studies. Administration of 100 mg doxycycline daily for 6 weeks resulted in the depletion of *Wolbachia* bacteria from *O. volvulus*, inhibition of the embryogenesis, long-term amicrofilaridermia, and killing of the adult worms [11]. Subsequent clinical trials demonstrated that doxycycline is also effective in depleting *Wolbachia* from bancroftian filariasis patients, leading to a 96% *Wolbachia* reduction and a 99% reduction in MF levels one year after treatment with 200 mg doxycycline for six weeks [60]. Combination therapy with doxycycline and a single dose of IVM resulted in a complete amicrofilaremia in bancroftian patients [60]. Further studies in LF patients showed that doxycycline regimens of 200 mg daily for 6 weeks followed by a single administration of IVM and ALB four months after doxycycline treatment not only reduced *Wolbachia* levels, but also had a macrofilaricidal effect of 89% after 24 months, determined by the absence of the filarial dance sign in the scrotum of male patients [61]. Lymphedema significantly improved after doxycycline administration already after 12 months [61]. Importantly, treatment duration of doxycycline could be reduced to 4 weeks with a single IVM dose 4 months after doxycycline regimen and still resulted in a macrofilaricidal activity of 83% after 24 months, which is comparable with the results of a 6-week doxycycline treatment [62]. Reduced treatment durations of three weeks with 200 mg doxycycline daily and a single DEC dose (6 mg/kg) were sufficient to completely ablate microfilaremia, but did not affect the adult worm burden [63].

For onchocerciasis it was shown that a 6-week doxycycline regimen with 100 mg daily resulted in a gradual decline of *Wolbachia* from month two to six following treatment, while the embryogenesis was inhibited by 6 months and MF declined by 11 months after treatment start. These effects were maintained for 18 months, whereas isolation of the adult worms through nodulectomy and collagenase digestion showed that only macrofilaricidal efficacy was observed at that time point and many worms were still viable [64]. A macrofilaricidal effect was observed for a 6-week therapy with 200 mg doxycycline after 20 and 27 months, resulting in the death of ~60% of the female adult worms as assessed by histological staining determining calcification, loss of body wall integrity, and absence of cathepsin D-like lysosomal aspartic protease of *O. volvulus* [65]. If newly acquired worms were subtracted, macrofilaricidal efficacy amounted to 70% [66]. Reducing treatment duration to 5 weeks and doxycycline concentration to 100 mg daily had a moderate macrofilaricidal effect with 51% viable adult female worms compared to 84% viable adult female worms in the control group, although the surviving female worms were not fertile [67]. Interestingly, combination treatment of doxycycline and IVM caused a quick decline in MF. However, unlike IVM treatment alone, the combination caused a permanent clearance of microfilarial loads in accordance with the inability of sterilized female worms to produce MF. Additional studies analyzing treatment duration and concentration revealed that 200 mg of doxycycline has to be given for four to six weeks, while two weeks treatment failed to deplete *Wolbachia* and MF [68]. Four- to six-week treatment with 200 mg doxycycline also resulted in a macrofilaricidal effect of 50 and 60%, respectively [65]. Moreover, a 6-week doxycycline treatment is also safe in areas co-endemic for loiasis, since *L.* *loa* has no *Wolbachia* bacteria, and doxycycline treatment does not bear the risk of life-threatening serious adverse events in *L. loa* patients with high microfilarial loads, unlike current MDA treatments with DEC and/or IVM [69], [70], [71].

Based on these results, it is recommended that doxycycline is given daily for 4 weeks at 200 mg [65], or for 5 weeks at 100 mg to achieve sustained sterilization of female adult worms and amicrofilaridermia in onchocerciasis patients [67]. To achieve the strongest macrofilaricidal effect, 200 mg doxycycline should be given daily for six weeks [72]. LF is treated with 200 mg doxycycline daily for four weeks to obtain a macrofilaricidal effect, while the reduction of lymphedema and hydrocele pathology can be achieved following a 6-week treatment of 200 mg doxycycline [73], [74].

In summary, these studies showed that doxycycline treatment depletes *Wolbachia* bacteria from the filariae causing LF and onchocerciasis, resulting in the inhibition of the embryogenesis, MF clearance over time, and a slow death of the adult worms. This is particularly advantageous since the treatment with doxycycline does not bear the risk of serious adverse events observed for onchocerciasis patients treated with DEC, or for loiasis patients with high MF loads treated with IVM, which is caused by the inflammation induced by the fast death of MF [70], [75], [76]. Thus, doxycycline administration is safe in areas co-endemic for those diseases overcoming the limitations of the current MDA. The results also show that the filariae causing LF (*Brugia* spp. and *W. bancrofti*) are more susceptible to the treatment with doxycycline compared to *O. volvulus*, since a 4-week treatment with doxycycline results in a long-lasting reduction in MF loads and adult worm removal in LF patients.

However, doxycycline treatment is contraindicated in pregnant and breast-feeding women, as well as children under the age of eight [74]. Furthermore, it requires daily treatments over several weeks and thus, it is rather used as an individual therapy. Doxycycline is administered by doctors in outpatient clinics in endemic countries with a health care system that provides individual care for filariasis, and in non-endemic countries like Europe and the US. Moreover, individual doxycycline treatment is recommended as an end-game strategy by the WHO to clear remaining onchocerciasis disease foci in Brazil and the Bolivarian Republic of Venezuela [77].

Next to human filariasis, canine heartworm disease caused by *D. immitis* is frequently treated with the standard monthly IVM (6 µg/kg), in combination with one round of doxycycline (10 mg/kg bi-daily) given for 4 weeks, followed by intramuscular injections with melarsomine (2.5 mg/kg) on day 60, 90 and 91 as recommended by the American Heartworm Society. This regimen leads to adult worm death, reduces MF loads, and prevents the aggravation of pulmonary damage in infected dogs [78].

## New macrofilaricidal drugs in clinical studies

### Drugs already tested in phase I clinical trials

#### High-dose rifampicin

Due to the limitations of doxycycline, additional antibiotics are under investigation for the treatment of filariasis. Rifampicin is of particular interest since it can be administered to children, and in initial preclinical tests, rifampicin depleted *Wolbachia* endosymbionts of *L. sigmodontis*, and reduced the filarial development and adult worm survival [79]. In first human clinical studies, 10 mg/kg/day rifampicin administered for two and four weeks to onchocerciasis patients resulted in impaired filarial embryogenesis and a *Wolbachia* reduction in female adult worms 18 months after treatment start. This was comparable to a 6-week treatment of doxycycline with 100 mg daily [80]. However, 78–100% of adult worms remained viable. Subsequent pharmacokinetic-pharmacodynamic (PK-PD) analysis of a dose-escalation study in *B. malayi*- and *O. ochengi*-infected mice showed that 10 mg/kg/day rifampicin (bioequivalent dose of 600 mg/day dose in humans) is suboptimal. Rather a bioequivalent human high-dose of 30–35 mg/kg/day is required to achieve a *Wolbachia* reduction of above 90% after 7 and 14 days in the *B. malayi* and *O. ochengi* model, respectively [81]. Thus, high-dose rifampicin has the potential to reduce the treatment duration to 1–2 weeks for human filariasis, and initial clinical studies for treatment of tuberculosis with rifampicin have shown that 20 and 35 mg/kg/day is safe and does not bear the risk of increasing toxicity in comparison to 10 mg/kg/day [82], [83], [84]. However, the number of patients in these studies is still limited, and larger patient cohorts with about 2000 patients will be required to ensure a sufficient safety profile, and are a prerequisite to be registered for filarial indication [85]. Additional phase II clinical studies using high-dose rifampicin to treat human filariasis are now under preparation and will investigate the safety profile of high-dose rifampicin in a larger study population [86], [87].

Of note, due to the short treatment duration of 1–2 weeks of rifampicin, drug resistance by *Mycobacterium* *tuberculosis* (TB) is not expected. However, possible risk for drug resistances in areas co-endemic for TB and filarial diseases cannot completely be excluded.

#### ABBV-4083

The Tylosin A analogue ABBV-4083 is a macrolide antibiotic with an improved pharmacokinetic profile with increased oral bioavailability and potency against *Wolbachia* [88], [89]. Using the* B. malayi*, *L. sigmodontis*, and *O. ochengi* animal models, ABBV-4083 showed a superior activity against *Wolbachia* compared to doxycycline. These preclinical studies demonstrated a *Wolbachia* depletion above 90% with blocked filarial embryogenesis and microfilarial release after treatment with ABBV-4083 for 1–2 weeks [88], [89]. Additional experiments in the *L. sigmodontis* rodent model showed that ABBV-4083 induced *Wolbachia* depletion as soon as 3 days after treatment start, and *Wolbachia* depletion continued in the following weeks after treatment ended [90]. Up to four missed treatments with ABBV-4083 did not impair the efficacy in depleting *Wolbachia* bacteria from* L. sigmodontis*, as long as the full regimen was subsequently completed [90]. Assessment of the safety of ABBV-4083 was done in preclinical and phase I clinical studies, which supports the progression of ABBV-4083 to phase II clinical studies [91], [92]. Thus, ABBV-4083 represents a next-generation macrofilaricidal oral drug candidate for the treatment of human filarial diseases, which may allow treatment regimens of 14 days or less. It has completed clinical phase I evaluation and phase II trials in onchocerciasis patients of the Democratic Republic of Congo begun in June 2021 [93].

In addition, in preclinical studies, using ABBV-4083 with ALB was tested in *L. sigmodontis*- and *B. pahangi*-infected jirds, and indicated that lower doses and shorter treatment durations of ABBV-4083 are possible when administered together with ALB [94]. *Wolbachia* depletion was improved by the combination therapy, which resulted in maintained clearance of peripheral MF loads compared to ALB and ABBV-4083 single treatments [94].

#### Oxfendazole as direct acting macrofilaricide

Oxfendazole belongs to the class of benzimidazoles, and is a broadspectrum anthelminthic veterinary drug that targets the β-tubulin of helminths. Thus, it was shown that oxfendazole is efficacious against *Taenia* *solium* in infected pigs [95]. Similar to flubendazole, oxfendazole has a high efficacy against adult worms when administered subcutaneously [96], [97], [98], [99], but unlike flubendazole, oxfendazole has an improved oral bioavailability [99], [100], [101]. However, flubendazole showed teratogenicity and aneugenicity [100], [102], which lead to the stop of further development of flubendazole as antifilarial drug in humans. For oxfendazole, indications of such a teratogenicity and aneugenicity were not reported, as oxfendazole exhibited no in vitro toxicity in the AMES assay, and the mouse lymphoma assay as well as in the in vivo rat micronucleus assay. Toxicity and behavioral studies in rats and cardiovascular studies in dogs further showed no safety concerns for oxfendazole [103]. Furthermore, a small study conducted in pregnant sows did not identify a risk of oxfendazole treatment on sows or the newborn pigs [104]. Subsequent first in human studies confirmed the safety of a multiple ascending dose [105], [106], [107]. However, since oxfendazole as well as ALB, which is currently used in MDA against LF, belong to the same chemical class as flubendazole, similar toxicity cannot be completely ruled out in humans and may require additional precautionary measures such as the exclusion of pregnant women and the usage of contraception for women of potential bearing age.

For filariae, in vitro studies have shown that oxfendazole inhibits motility of *O. gutturosa* adult worms, *O. volvulus* pre-adults (L5), and *O. lienalis* MF [99]. Preclinical studies provided further evidence that a five-day regimen of orally administered oxfendazole killed all adult worms in the *L. sigmodontis* mouse model [99]. Using the *L. sigmodontis* jird model, the effect on microfilaremia was analyzed. A 10-day regimen of oxfendazole inhibited filarial embryogenesis and resulted in a slow and continuous decline of peripheral MF, and finally to a complete clearance of MF [99]. Importantly, the decline in MF resulted from the inhibited embryogenesis rather than from a direct microfilaricidal effect [99]. Based on the animal pharmacokinetic studies, the human efficacious dose is predicted to range from 1.5–4.1 mg/kg [99]. A first human multiple ascending dose study with 3, 7.5, and 15 mg/kg oxfendazole daily for a total of five days has already been completed [105], [106] and has shown no adverse reactions [107]. Since the predicted human efficacious dose is within this tested and well-tolerated range, oxfendazole has the potential to be a potent drug candidate that could be used for case management and to treat remaining foci of onchocerciasis. Furthermore, as oxfendazole had no strong microfilaricidal efficacy in the *L. sigmodontis* model [99] and in mice infused with *L. loa* MF [108], it may also present a promising candidate for the treatment of loiasis. Thus, oxfendazole is currently under consideration for phase II clinical studies, and within the HELP consortium of the European Union’s Horizon 2020 activities [109], oxfendazole bioavailability studies and field-applicable tablet formulations are prepared [110]. However, it still has to be determined if oxfendazole is safe for treatment in neurocysticercosis patients, as the death of *T. solium* cysticerci in the brain following treatment with the benzimidazole ALB induces inflammatory responses and temporarily worsens neurological symptoms [111]. Since ALB or praziquantel therapy in general can provoke inflammatory responses upon dying cysts, co-administration of corticosteroids in neurocysticercosis patients are frequently required [112]. Therefore, onchocerciasis and cysticercosis co-endemic areas may require a test and treat strategy for oxfendazole treatment.

#### Emodepside

Repurposing of drugs which are already registered for animal health has been one of the focuses to identify new macrofilaricidal candidates against filariasis. Next to oxfendazole, emodepside is a drug frequently used in veterinary settings to treat parasitic gastrointestinal nematodes including roundworms, hookworms, and strongyloides [113], [114]. Emodepside belongs to the cyclooctadepsipeptides, and PF1022A is the first anthelminthic member recognized of this class, which is a compound isolated from the fungus *Rosellinia* spp. [115]. Emodepside acts on the Ca^2+^-gated K^+^-channel SLO-1 of nematodes [116]. In vitro studies have shown that emodepside paralyses the different larval stages of several filariae, including *A. vitae*, *Brugia* spp., *D. immitis*, *L. sigmodontis*, and *Onchocerca* spp. [117]. Importantly, adult worms seemed to have the highest in vitro sensitivity towards emodepside compared to the different larval stages [117]. Furthermore, emodepside was highly efficacious in rapidly decreasing microfilarial loads of *L. sigmodontis*, *A. viteae*, and *B. malayi* in infected *Mastomys coucha* in vivo. A single dose of 3.125 mg/kg (*L. sigmodontis*) and 6.25 mg/kg (*B. malayi*) reduced the microfilarial loads by more than 95% after 3 days already. Treatment with a single dose of 100 mg/kg and five treatments with 100 mg/kg emodepside caused a macrofilaricidal effect in *A. vitae*- and *L. sigmodontis*-infected animals, respectively, while no effect on adult worms was observed for *B. malayi* [118]. Even though *Brugia* spp. were least susceptible to emodepside, *O. gutterosa* and *O. lienalis* were paralyzed and killed even at low doses of emodepside [119]. A proof of principal study in *O. ochengi*-infected cattle confirmed that a 7-day emodepside treatment rapidly clears MF, inhibits filarial embryogenesis, and mediates a macrofilaricidal efficacy after 18 months [120]. Thus, emodepside targets multiple life-cycle stages of filariae, and due to the high susceptibility of adult worms of the *Onchocerca* spp., it is under investigation for the treatment of onchocerciasis [115]. First clinical human trials have demonstrated the safety of emodepside, and a phase II clinical trial in onchocerciasis patients in Ghana has started in January 2022 [121]. However, targeting several stages of the filariae including the MF stage could bear the risk for adverse events in patients with high MF loads, and may argue against its use in loiasis co-endemic areas. On the other hand, emodepside may also be active against intestinal helminths and may therefore present a pan-nematode candidate that tackles both filarial and intestinal helminth infections [115].

#### Auranofin

Another re-purposed drug to treat filarial diseases is auranofin (2,3,4,6-tetra-O-acetyl-1-thio-beta-D-glucopyranosato-S (triethylphosphine) gold), which is an FDA-approved drug used to treat rheumatoid arthritis in humans [122], [123]. Next to auranofin’s activity against *Brugia* spp. and *O. ochengi* MF, it showed an even higher activity against adult worms of *Brugia* spp. and *O. ochengi*, as well as against L3 larvae of* O. volvulus* in vitro. Since highest concentrations were required to inhibit *L. loa* MF, auranofin may provide a drug which can be administered in areas co-endemic for *L. loa*. In vivo, 28-day treatment with 5 mg/kg auranofin twice a day on weekdays reduced adult worm burden of *B. malayi*-infected jirds by 91% compared to the vehicle control group. Mode of action of auranofin is mediated through the inhibition of the thioredoxin reductase [124] and the related thioredoxin glutathione reductase [125]. A first phase I clinical trial was conducted, which reported auranofin’s safety [126].

### Drugs in preparation for phase I clinical trials

#### Corallopyronin A

Corallopyronin A (Cor A) is an α-pyrone ring-containing natural product from *Corallococcus* *coralloides* [127], and it is a non-competitive inhibitor of bacterial DNA-dependent RNA polymerase by targeting the switch region rather than the active site [128], [129], [130], [131]. It is highly effective against Gram-positive bacteria including rifampicin-resistant *Staphylococcus* *aureus*, but has low efficacy against Gram-negative bacteria unless genes for the *tolC* or other efflux pumps are missing [130]. Even though Gram-negative bacteria are typically not susceptible, Cor A showed in vitro and in vivo activity against *Wolbachia* bacteria [132]. Since *Wolbachia* bacteria have a significant reduced genome, the efflux pump pathways are incomplete and they are unable to produce lipopolysaccharide, which renders them susceptible to Cor A [38].

In the *L. sigmodontis* model, Cor A significantly reduced *Wolbachia* bacteria, completely cleared microfilaremia and it has shown a robust reduction of adult worm burden when administered alone or in combination with ALB [133]. Safety and toxicity tests in vitro and in vivo suggest that Cor A is safe and non-toxic, and its progress towards first clinical studies, supported by the German Center for Infection Research (DZIF) and the EU Horizon 2020 HELP consortium, is under preparation.

#### AWZ1066S

A highly selective and potent anti-*Wolbachia* candidate is the azaquinazoline AWZ1066S, which showed a maximal clearance of *Wolbachia* within one day of in vitro drug exposure. Thus, *Wolbachia* clearance in vitro of AWZ1066S is much faster, and thus superior compared to doxycycline and rifampicin. AWZ1066S reduced more than 98% of *Wolbachia* in the *B. malayi* SCID mouse and *L. sigmodontis* gerbil model and inhibited embryogenesis in the latter [134]. Furthermore, beginning six weeks post AWZ1066S treatment in *L. sigmodontis*-infected gerbils, a continuous decline in peripheral MF was observed, and after 14 weeks post treatment start, MF were completely cleared. Similar to all anti-Wolbachials, AWZ1066S rather inhibits the embryogenesis than having a direct microfilaricidal effect [134]. Preclinical safety has already been proven, and human PK simulation predict a *Wolbachia* reduction by more than 90% in more than 90% of the patients after a 7-day regimen with 10 mg/kg AWZ1066S [134]. Due to this potent anti-Wolbachial efficacy and the early assessment of its safety, AWZ1066S fits the criteria for the target product profile for novel drug candidates for human filariasis, and first clinical studies to assess the safety of AWZ1066S are intended [135].

#### CC6166

CC6166 is a direct-acting compound developed by Celgene (now Bristol-Myers Squibb), which is supported by the DND*i*, and it is currently under investigation for its macrofilaricidal activity [136], [137], [138].

### Backup candidates on hold

#### AN11251

The boron-pleuromutilin AN11251 exhibited a solid potency against *Wolbachia* in vitro, and thus was targeted for further preclinical in vivo studies. Using the *L. sigmodontis* mouse model, AN11251 exhibited a good oral bioavailability, depleted more than 99% of *Wolbachia* after 10 to 14 days of treatment, and is thus superior to the human bioequivalent dose of doxycycline [139], [140]. Preliminary in vitro and in vivo safety assessment support further evaluation of AN11251 as a preclinical anti-*Wolbachia* candidate for human filarial diseases.

#### CBR417/CBR490 

Further preclinical candidates on hold are the quinazolines CBR417 and CBR490, which showed a potent and selective anti-*Wolbachia* activity. Ex vivo assays demonstrated superior *Wolbachia* depletion by CBR417 and CBR490 from female *B. pahangi* ovaries compared to doxycycline. In vivo, these quinazolines rapidly cleared *Wolbachia* in the *L. sigmodontis* mouse model, and treatments as short as 4 days with 60 mg/kg/day eliminated more than 99% of *Wolbachia* in *L. sigmodontis* adult female worms. Even one single dose regimen or two doses within two weeks were sufficient to reduce more than 99% of *Wolbachia* [141]. Thus, these two new compounds highlight the potential of short-regiments using anti-*Wolbachia* drug candidates.

## Summary and outlook

Current treatment strategies for onchocerciasis and LF face several limitations preventing the successful elimination of those diseases. The biggest challenge is still the lack of a macrofilaricidal drug that is safe and requires only short treatment durations, and thus would vastly accelerate the elimination of these debilitating diseases. By depleting *Wolbachia* bacteria, doxycycline was the first well-tolerated macrofilaricidal drug and paved the path for the identification of new anti-Wolbachial drugs with macrofilaricidal activity. Safety of high-dose rifampicin was provided in clinical studies for tuberculosis, and preclinical studies showed promising results for high-doses of rifampicin to be an effective anti-Wolbachial antibiotic with shorter treatment durations and a safe administration of rifampicin in children, thus overcoming two limitations of doxycycline. ABBV-4083, Cor A, and AWZ1066S are additional promising anti-Wolbachial drug candidates that could allow shorter treatment regimens. Based on preclinical modeling, suggested treatment regimens of 7 or 14 days are possible with ABBV-4083, which completed the clinical phase I and is currently under phase II evaluation. Cor A is the first published anti-*Wolbachia* candidate that significantly reduced the *L. sigmodontis* adult worm burden by depleting *Wolbachia* bacteria in preclinical studies. In vitro, AWZ1066S showed superior *Wolbachia*-depletion compared to doxycycline and rifampicin, and in vivo preclinical studies presented a very fast *Wolbachia*-reduction, which will enable short treatment durations predicted to be of 7 days or less.

Promising direct-acting macrofilaricidal candidates currently under preparation for or in phase II clinical studies are oxfendazole and emodepside, respectively. Oxfendazole presented a robust macrofilaricidal but no strong microfilaricidal activity in preclinical studies with* L. sigmodontis*, and based on first human clinical trials, the predicted human efficacious dose for oxfendazole appears to be safe. Emodepside on the other hand targets multiple life-cycle stages of filariae, and first clinical human trails have demonstrated the safety of emodepside. Based on its broad activity against both filarial and intestinal nematodes, it is potentially useful for several nematode diseases [115], [117]. Next to emodepside, CC6166 is supported by the DND*i* and currently investigated for its macrofilaricidal activity. Furthermore, several further candidates such as AN11251 and CBR417/CBR490 are currently on hold and serve as backup drugs (Table 1 (Tab. 1)).

Recently, the FDA approved moxidectin as a treatment for onchocerciasis, and with its superior activity against MF in comparison to IVM, it may replace IVM, and thus reduce transmission and infection rates for onchocerciasis. Moxidectin could be especially effective in areas with persistent transmission despite high IVM coverage due to substantial inter-treatment transmission [142]. However, moxidectin still faces the same problems as the other microfilaricidal drug candidates. Moxidectin has the advantage that it only requires a single annual treatment, while the macrofilaricidal candidates are expected to require regimens of 7 to 14 days, and thus are more labor-intensive and expensive in comparison to moxidectin. However, selective macrofilaricides and anti-Wolbachials should be safe for treatment in areas co-endemic for loiasis, whereas moxidectin, similar to IVM, may cause severe adverse events in loiasis patients with high MF numbers. In particular, ABBV-4083 and oxfendazole are quite advanced in the development and may arise as good candidates to substantially eliminate onchocerciasis. However, registration of new macrofilaricidal drugs is planned but will not be done in the next few years, which reduces the time to reach the goals of the WHO roadmap. What will be more difficult is the distribution of the new drugs, since it will rely on a strong pharmaceutical partner. This partnership is lacking for all new macrofilaricidal drug candidates including moxidectin. Therefore, new drugs will require partnering for further development, and will mostly impact the outcome if onchocerciasis will be eliminated in the near future. Most candidates have been developed in collaboration with pharmaceutical companies, academia, and non-profit organizations. The research has been supported by programs such as the Bill & Melinda Gates foundation and TDR (Special Programme for Research and Training in Tropical Diseases), which recently have stopped the funding for additional preclinical development. Even though several candidates have been identified and tested in first clinical studies, Cor A, AWZ1066S, and CC6166 have not been tested in humans, and thus the safety profile is still preliminary. Therefore, these safety data could still pose the risk of a high attrition rate and limit the number of drugs in the pipeline for the treatment of onchocerciasis. Moreover, further development of the preclinical candidates AN11251 and CBR417/490 has been stopped and placed the candidates on hold due to lack of finance. Thus, due to the high attrition rate of drugs entering the first clinical phases and even among candidates that successfully cleared phase I clinical studies, a healthy drug pipeline is required. Despite the backup candidates that are currently on hold for the development, effective elimination of onchocerciasis should not be focused on a single candidate, but several drugs should be developed that address the specific need of the patients depending on its context. Such a pipeline could also be used to identify improved drugs that enable e.g. pediatric formulations, treatments for pregnant and lactating women, and a safe macrofilaricidal treatment for loiasis patients.

## Notes

### Competing interests

The authors declare that they have no competing interests.

## Figures and Tables

**Table 1:**
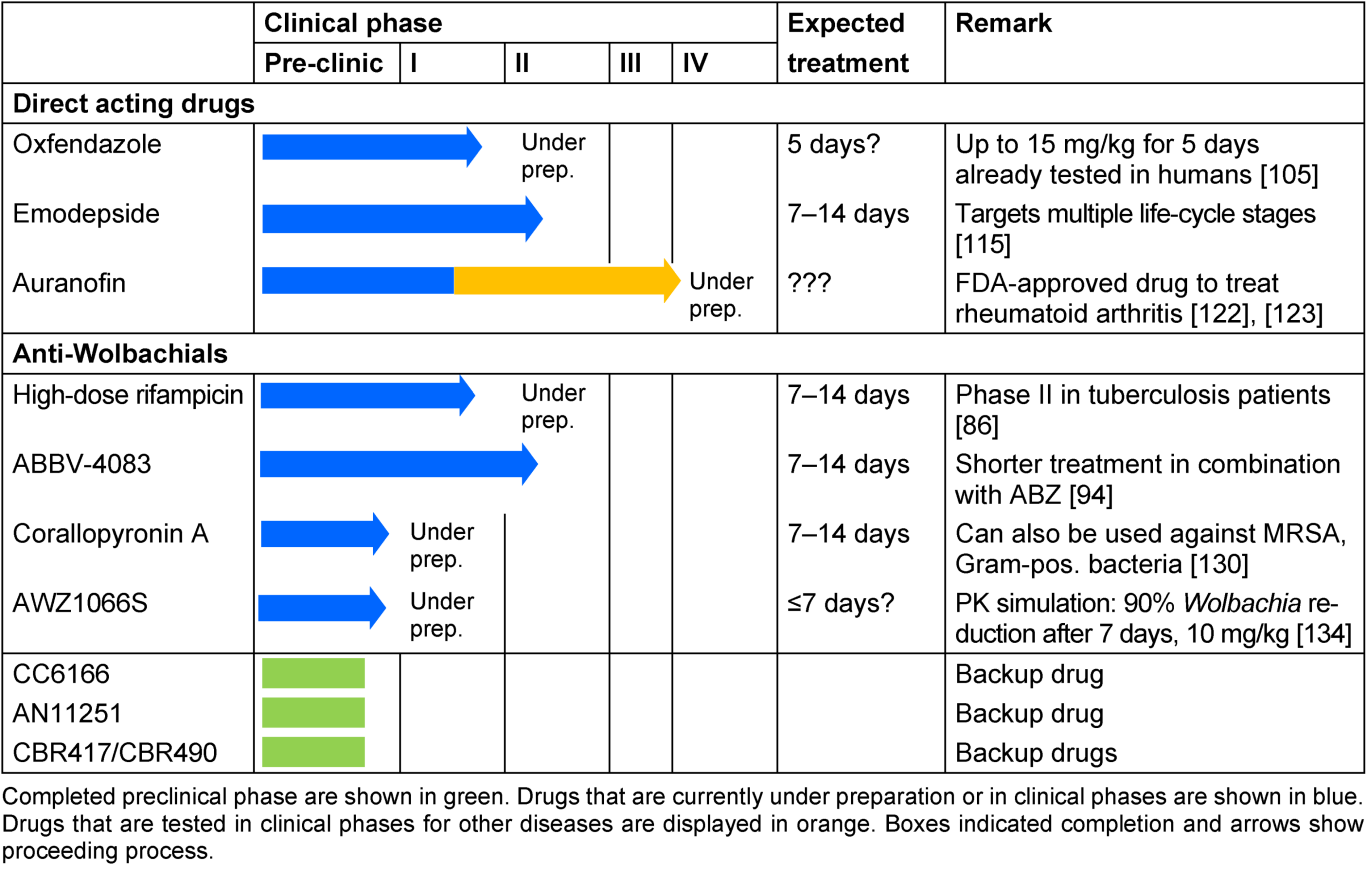
Summary of new macrofilaricidal candidates and their current clinical status
